# Functional Polymorphism in the Interleukin 6 (*IL6*) Gene with Respect to Depression Induced in the Course of Interferon-α and Ribavirin Treatment in Chronic Hepatitis Patients

**DOI:** 10.1007/s00005-016-0441-7

**Published:** 2017-01-12

**Authors:** Dorota Frydecka, Tomasz Pawłowski, Dariusz Pawlak, Krzysztof Małyszczak

**Affiliations:** 1 Department and Clinic of Psychiatry, Wroclaw Medical University, Pasteura 10, 50-367 Wroclaw, Poland; 2 Department of Pharmacodynamics, Medical University in Bialystok, Białystok, Poland; 3 Division of Psychotherapy and Psychosomatic Medicine, Department and Clinic of Psychiatry, Wroclaw Medical University, Wroclaw, Poland

**Keywords:** Chronic hepatitis C, Depression, Interleukin-6, Functional genetic polymorphism, Longitudinal study, Hepatitis C virus

## Abstract

Interleukin (IL)-6 is a multifactorial cytokine known to be increased in patients with chronic hepatitis C (CHC) and to be predictive of depression incidence. The aim of the study was to explore the association between *IL6* gene C-174G polymorphism and depressive symptom severity in the longitudinal study design following the course of pegylated interferon/ribavirin treatment in CHC patients. In our study, we included 62 CHC subjects. They were assessed using present state examination, Beck Depression Inventory (BDI) and Montgomery Åsberg Depression Rating Scale (MADRS) at weeks 0, 3, 5, 9, 13, 24 and 24 weeks after the end of treatment. The risk of depression was associated with higher baseline MADRS score and BDI score. Interestingly, when stratified by *IL6* C-174G polymorphism, higher baseline depressive symptom severity measured by MADRS and BDI predicted higher risk of depression in the course of antiviral treatment only in high IL-6 producers—G allele carriers (patients with GG and CG genotypes) (*p* = 0.004, *p* = 0.00008, respectively). There is interaction between severity of baseline depressive symptoms at the beginning of antiviral therapy and *IL6* gene C-174G polymorphism leading to increased risk for the development of depressive episode in CHC patients in the course of antiviral treatment.

## Introduction

Interferon (IFN)-α is the main pharmacological treatment for chronic hepatitis C virus (HCV) infection, because of its potent antiviral, antiproliferation and immunomodulatory properties (Lamers et al. 2012); however, at the same time IFN-α is known to induce several neuropsychiatric side effects, including anorexia, fatigue, lethargy, loss of interest, lack of concentration, irritability, cognitive decline, emotional lability and social withdrawal (Cattie et al. 2014). Depression is a particularly common side effect and in some rare cases may be associated with deliberate self-harm or suicide attempts. Thus, identification of risk factors that lead to depression may help to recognize patients at risk who may benefit from additional psychological support (Smith et al. 2011).

IFN-α induces depression, but only in a subset of patients (Capuron et al. 2002; Lotrich et al. 2007; Musselman et al. 2001) and several vulnerability factors that predict depression after IFN-α treatment have been described, such as: levels of circulating cytokines (Krueger et al. 2011), brain-derived neurotrophic factor serum level (Kenis et al. 2011), elevated ratio of arachidonic acid to long-chain omega-3 fatty acids (Lotrich et al. 2013), changes in serotoninergic system (Bonaccorso et al. 2002), hypothalamic–pituitary–adrenal axis deregulation and glucocorticoid resistance (Raison et al. 2010).

Most of the published genetic association studies of mood disorders have focused on functional polymorphisms in the loci encoding the serotonin transporter (*SLC6A4*), serotonin 2A receptor (*5HTR2A*), tyrosine hydroxylase (*TH*), tryptophan hydroxylase 1 (*TPH1*), and catechol-*o*-methyltransferase (*COMT*); however, recent meta-analysis of 183 papers that studied 393 polymorphisms in 102 genes found significant evidence for association for the following genes: APOE, DRD4, GNB3, MTHFR, SLC6A3 and SLC6A4 (Lopez-Leon et al. 2008).

It has also been shown that biological mechanisms responsible for IFN-α-induced depression can also be influenced by underlying genetic vulnerability. Several genetic polymorphisms have been analyzed to identify increased risk factors for developing depression in the course of IFN-α treatment, including serotonin transporter gene (*5*-*HTTLPR*) (Bull et al. 2009), 5-HT1A receptor gene (Kraus et al. 2007), tryptophan hydroxylase-2 gene (*TPH2*) (Kraus et al. 2007), phospholipase A2 (*PLA2*) and cyclooxygenase 2 (*COX2*) gene (Su et al. 2010). Additionally, with respect to immune system the following genes have been analyzed: IFN-α/β receptor 1 (*IFNAR1*) gene (Smith et al. 2012), IL-6 gene (*IL6*) (Bull et al. 2009; Udina et al. 2013), IL-1 α and β (*IL1A, IL1B*) genes (Smith et al. 2012), IFN-γ (*IFNG*) gene (Oxenkrug et al. 2011) as well as genes encoding IL28B (Pasha et al. 2013), TGF-β1 (Pasha et al. 2013) and TNF-α (Pasha et al. 2013). Moreover, recently 15 genes were identified that were selectively hyper-responsive to exogenous IFN-α in patients that developed depressive side effects (Schlaak et al. 2012).

The majority of the studies focus on genetic vulnerability to neuropsychiatric disturbances in the course of IFN-α treatment based on the comparison between the patients who developed depression with the non-depressed group. However, little is known about the influence of genetic polymorphisms on the symptom severity in the longitudinal approach that allows for multiple assessment of psychological functioning from the beginning to the end of treatment in all patients undergoing treatment, and not only patients who ultimately develop depression.

## Materials and Methods

### Subjects

We included 62 subjects in the study. All subjects had chronic HCV infection with compensated liver disease. Subjects were recruited from the Department of Infectious Diseases, Hepatology and Acquired Immunodeficiencies Medical University in Wroclaw, Poland. All subjects were of Caucasian origin (31 females and 31 males of mean age 49.16 ± 10.73 and 33.55 ± 11.49, respectively). They were entirely native unrelated Polish population recruited from the same geographic area—Lower Silesia region. All patients were receiving pegylated IFN-α2a (Pegasys; Hoffmann-LaRoche, Basel, Switzerland) at a dose of 180 µg once per week. Additionally, they were receiving ribavirin (Schering-Plough Corp) at a dose of 1000 mg per day if their body weight was <75 kg or 1200 mg per day if their body weight was ≥75 kg. The duration of treatment was 48 weeks, except for patients infected with genotype 3 HCV who were treated for 24 weeks.

Patients having a history of traumatic brain injury or neurologic disorders were excluded from the study by detailed medical examination. Other exclusion criteria included prior treatment with IFN-α therapy, pregnancy, autoimmune disorder and any cause for liver disease other than HCV infection. None of the participants were receiving antidepressant or anxiety medications 6 months prior to inclusion into the study. There were no subjects addicted either to drugs or alcohol in our study sample. The study was approved by the Institutional Ethics Committee and all participants provided written informed consent prior to participation.

### Study Design

Subjects had a baseline psychiatric evaluation of mental state and previous psychiatric history, using present state examination (PSE) from Schedules of Clinical Assessment in Neuropsychiatry (SCAN 2.0) performed by a senior board-certified investigator. Categorical depressive episode during therapy was diagnosed according to Diagnostic and Statistical Manual IV Axis I Disorders. Additionally, Montgomery Åsberg Depression Rating Scale (MADRS) and Beck Depression Inventory (BDI) were used to assess the severity of depressive symptoms. The study followed a prospective longitudinal cohort design. Subjects were evaluated with PSE, MADRS and BDI before they started treatment (week 0) and at weeks 3, 5, 9, 13 and 24, at the end of treatment as well as 24 weeks after the end treatment. At each assessment point blood samples were also collected. Preliminary results on depressive symptoms during treatment with IFN-α for HCV infection have been published previously (Malyszczak et al. 2006).

### Genotyping

Genomic DNA was prepared from peripheral white blood cells from whole frozen blood using the QIAamp DNA Blood Mini Kit (Qiagen GmbH, Hilden, Germany). Single nucleotide polymorphism in *IL6* gene (rs1800795) was genotyped by a single specific primer-polymerase chain reaction using OneLambda commercial sets PCytgen. As a quality control, we retyped 10% with allelic discrimination methods using LightSNiP Assay on demand for rs1800975 (TIB MOLBIOL, Poznan, Poland).

### Statistical Analysis

Demographic and clinical data among patients with and without depressive episode in the course of antiviral treatment were compared using ANOVA test (age, illness duration, total IFN dose, total ribavirin dose, treatment duration, number of copies of the HCV, grading/staging of disease, TSH level) and *χ* ^2^ test (gender, HCV, genotype, family history of psychiatric illness, past history of psychiatric illness). Evaluation of the Hardy–Weinberg equilibrium (HWE) was performed by comparing the observed and expected genotype distribution using the *χ* ^2^ goodness-of-fit test. Distribution of alleles and genotypes of *IL6* C-174G gene polymorphism was assessed using *χ* ^2^ test. Differences between depression severity assessed with MADRS and BDI scales at the beginning of the treatment, during the treatment and 24 weeks after completion of treatment were compared using paired samples *t* test. Severity of baseline depressive symptoms measured with MADRS and BDI scales with respect to the *IL6* C-174G gene polymorphism was compared using Mann–Whitney *U* test. Differences were considered as statistically significant if the two-tailed *p* value was less than 0.05. All analyses were performed using the Statistical Package for Social Sciences version 20.

## Results

**Fig. 1:**
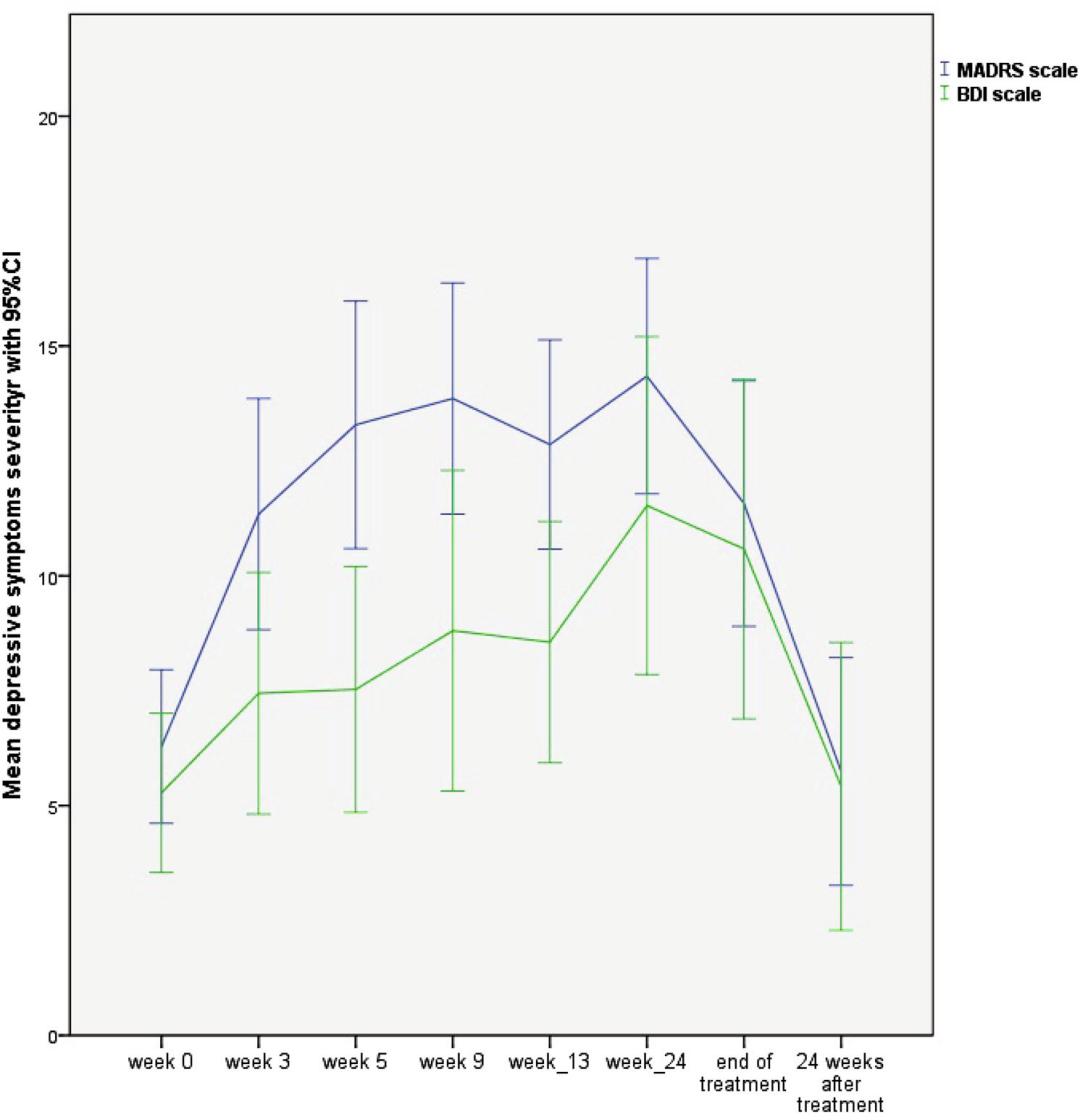
Depressive symptoms measured by MADRS and BDI scales during treatment and after 24 weeks after the end of treatment

In our study, we observed an inverted U-shaped curve of depressive symptom severity in the course of antiviral therapy (Fig. 1). There was a statistically significant difference between the depression severity at the beginning of the treatment as assessed by MADRS and BDI (6.39 ± 4.69 and 6.16 ± 5.557, respectively) and in the course of treatment, calculated as mean value of depressive symptoms in the course of treatment as assessed by MADRS and BDI (12.78 ± 6.68 and 9.51 ± 7.49, respectively; *p* < 0.00001). The depressive symptoms were diminished 24 weeks after the end of the treatment as assessed by MADRS and BDI (5.37 ± 6.35 and 4.75 ± 7.76, respectively; *p* < 0.0001) reaching the level of depression assessed at the beginning of treatment by MADRS and BDI (*p* = 0.25 and *p* = 0.11, respectively; Fig. 1).

In our study, 24 patients (38.71%) developed depression in the course of IFN-α and ribavirin therapy. Patients with and without depressive episode did not differ in age, gender, duration of illness, treatment parameters, virus genotype, grading and staging of disease, TSH level (*p* > 0.05; Table 1) or family history of psychiatric disorders (Table 2). However, we have found an association between development of depressive episode in the course of antiviral therapy and patients’ past psychiatric history of depressive or anxiety disorder (Table 2).

**Table 1:** Demographic and clinical characteristics of the patients with respect to IFN-α-related depressive episode

Demographic and clinical variables	Depressive episode in the course of treatment		*p* value
	No (*n* = 38)	Yes (*n* = 24)	
Age (years)^a^	47.79 ± 11.09	46.79 ± 11.80	0.73^c^
Gender [*n* (%)]			
Males	18 (47.4%)	13 (54.2%)	0.60^d^
Females	20 (52.6%)	11 (45.8%)	
Duration of illness (years)^a^	6.84 ± 4.06	7.42 ± 4.10	0.59^c^
Total interferon dose (mg)^a^	6640 ± 2113	6700 ± 2130	0.91^c^
Total ribavirin dose (mg)^a^	291505 ± 97937	281042 ± 102149	0.69^c^
Treatment duration (weeks)^a^	39.82 ± 11.81	38.71 ± 12.49	0.73^c^
HCV RNA (10^6^ copies/mL)^a^	7.4 ± 8.80	14.85 ± 24.10	0.09^c^
TSH level at baseline (mIU/L)^a^	1.20 ± 1.19	1.77 ± 1.24	0.08^d^
Virus genotype			
1	26 (68.5%)	16 (66.7%)	0.94^d^
3	11 (28.9%)	7 (29.1%)	
4	1 (2.6%)	1 (4.2%)	
Grading (Metavir scale)^b^	2.07 ± 0.68 (2)	1.91 ± 0.7 (2)	0.4^e^
Staging (Metavir scale)^b^	2.09 ± 0.70 (2)	1.75 ± 0.88 (1.5)	0.06^e^

^a^Mean ± standard deviation (SD)

^b^Mean ± SD (median)

^c^ANOVA test

^d^ *χ* ^2^ test

^e^Mann–Whitney *U* test

**Table 2:** History of psychiatric disorders and baseline depressive symptoms with respect to IFN-α-related depressive episode

Psychiatric evaluation	Depressive episode in the course of treatment		*p* value^a^
	No (*n* = 38) (%)	Yes (*n* = 24) (%)	
Family history of psychiatric disorder			
None	22 (57.9)	14 (58.3)	0.85
Depression	4 (10.5)	2 (8.3)	
Bipolar disorder	0 (0)	0 (0)	
Schizophrenia	1 (2.6)	1 (4.2)	
Anxiety disorder	2 (5.3)	1 (4.2)	
Alcohol dependence	8 (21.1)	5 (20.9)	
Suicide	1 (2.6)	1 (4.2)	
Past history of psychiatric disorders			
None	32 (57.89)	14 (58.33)	0.038
Depression	5 (36.84)	4 (20.83)	
Anxiety disorder	1 (5.26)	3 (12.5)	
Suicide attempts	0 (0)	3 (8.33)	

^a^ *χ* ^2^ test

The genotypic distribution of *IL6* gene C-174G polymorphism was in HWE. There were also no differences in the distribution of alleles or genotypes of *IL6* gene polymorphism between these groups of patients (*p* > 0.05; Table 3). Depressive symptom severity assessed with MADRS and BDI at the beginning of therapy were predictors of development of depressive episode during the course of antiviral treatment (*p* = 0.00004, *p* = 0.00003, respectively; Table 4). Interestingly, after stratifying patients according to the *IL6* gene C-174 gene polymorphism genotypes, we have shown that higher baseline depressive symptom severity measured by MADRS and BDI predicted higher risk of depression in the course of antiviral treatment in G only allele carriers (patients with GC and GG genotype) (*p* = 0.004, *p* = 0.00008, respectively; Table 4).

**Table 3:** *IL6* gene polymorphism with respect to IFN-α-related depressive episode

*IL6* gene C-174G polymorphism rs1800795	Depressive episode in the course of treatment		*p* value^a^
	No (*n* = 38)	Yes (*n* = 24)	
Genotype			
CC	5 (13.2%)	5 (20.8%)	0.17
CG	21 (55.3%)	12 (50.00%)	
GG	12 (31.5%)	7 (29.2%)	
Allele			
C	31 (40.8%)	28 (45.8%)	0.49
G	45 (59.2%)	32 (54.2%)	
Carriers			
G**−** [CC]	5 (13.2%)	5 (7.69%)	0.37
G+ [CG and GG]	33 (86.8%)	60 (92.31%)	

^a^ *χ* ^2^ test

**Table 4:** The association between baseline MADRAS scores and the risk of depressive episode in the course of antiviral treatment with respect to *IL6* gene C-174G polymorphism

*IL6* gene C-174G polymorphism rs1800795	Depressive episode in the course of treatment		*p* value^a^
	No (*n* = 38)	Yes (*n* = 24)	
MADRS score at the beginning of treatment			
Whole group	4.82 ± 3.94	9.93 ± 4.87	0.00004
G− [CC]	7.20 ± 4.76	9.20 ± 4.21	0.55
G+ [CG + GG]	4.45 ± 3.75	9.74 ± 5.13	0.00008
BDI score at the beginning of treatment			
Whole group	4.53 ± 5.33	8.96 ± 4.81	0.00003
G− [CC]	4.00 ± 5.34	11.00 ± 2.55	0.09
G+ [CG + GG]	4.61 ± 5.40	8.42 ± 5.17	0.004

^a^Mann–Whitney *U* test

## Discussion

Anti-HCV treatment causes a dramatic increase in the prevalence of depression, from 8% at the beginning to 38.71% in the middle of the treatment. The starting value of depression prevalence is almost threefold more than in the general population in Poland (3%) (Kiejna et al. 2015) and similar to the prevalence among university students in Wroclaw (9.8%) (Zagdanska and Kiejna 2016). The patient population is more depressive than the general population, so the prevalence difference between them is likely to be demonstrated. The study showed that treatment resulted in a significant increase not only in the symptoms, but also in the prevalence of depression.

IFN-α-induced neuropsychiatric symptoms have been attributed to the induction of pro-inflammatory cytokines that modulate several neurophysiological and neuroendocrine systems involved in mood regulation (Raison et al. 2006). IFN-α is a potent inducer of pro-inflammatory cytokines, including IL-6. This cytokine appears to play an important role in the development of psychopathological side effects including depressive symptoms as well as in viral response in the course of IFN-α treatment (Guzman-Fulgencio et al. 2012).

In our study, we have shown that patients with past history of depressive or anxiety disorders have higher risk of developing depressive episode during antiviral therapy. This result is in line with numerous studies on the general population (Patten 2013) as well as with a recent meta-analytic study on IFN-induced depression in CHC patients (Udina et al. 2012). Additionally, we have shown the role of baseline depressive symptom severity in the development of depressive episode in patients receiving a combination of pegylated IFN-α and ribavirin. This result is in line with several previous studies showing the role of sub-syndromal baseline symptoms in patients having antiviral therapy (Dell’Osso et al. 2013; Mahajan et al. 2014).

A functional G > C single nucleotide polymorphism (C-174G, rs1800795) in the promoter region of the *IL6* gene is associated with differential IL-6 expression and ultimately IL-6 plasma concentrations. It has been shown that the G allele is associated with higher plasma concentrations of IL-6 during immune activation than the C allele (Fishman et al. 1998). It has been shown that “high IL-6” genotype is associated with more depressive symptoms during IFN-α treatment in CHC patients (Bull et al. 2009; Udina et al. 2013). This finding is consistent with the evidence that plasma IL-6 positively is associated with depressive symptoms among patients without any immune-modulatory treatment (Liu et al. 2012) as well as among patients during IFN-α treatment (Bonaccorso et al. 2001; Prather et al. 2009; Wichers et al. 2007) including CHC patients (Udina et al. 2012). Moreover, baseline psychopathological symptom severity has been shown to predict mood episodes during CHC therapy (Dell’Osso et al. 2013; Rempel et al. 2014). In our study we have shown that even though *IL6* C-174G gene polymorphism was not associated with the risk of depression, there was an interaction between IL-6 high producer G allele carriers and baseline depressive symptoms, conferring together higher risk of depressive episode in the course of antiviral treatment in CHC patients. To the best of our knowledge, it is the first study showing this effect.

According to a recent meta-analytic study, one in four CHC patients who start IFN and ribavirin treatment develop an induced depressive episode (Udina et al. 2012). The results of our study point to the high importance of detailed psychiatric evaluation of CHC patients subjected to antiviral therapy, including past psychiatric history and baseline depressive symptom severity, since these are risk factors for the development of depressive episode in the course of treatment and might influence treatment outcome.

Our study has several limitations that need to be addressed. First of all, there was a relatively small sample size of our study group; however, it should be noted that each patient was evaluated in detail at eight time points over 48 weeks with not only PSE, but also MADRS and BDI. However, the small number of subjects precluded us from conducting further subgroup analyses, and therefore we cannot exclude the effect of differences in the pattern of use of psychotropic medications on depression scores. Additionally, our findings should be approached with caution, given the lack of concomitant biological measures, such as plasma and cerebrospinal fluid concentrations of IL-6. This might have enriched the results of our study, since we would have been able to confirm as to whether G allele carriers were indeed producing higher levels of IL-6 and assess the influence of IL-6 levels depressive symptom severity and risk of depression episode development in the course of antiviral treatment.

In conclusion, past psychiatric history and severity of baseline depressive symptoms at the beginning of antiviral therapy together with *IL6* C-174G gene polymorphism may serve as risk factors for the development of depressive episode in CHC patients.

## Abbreviation

HCV: Hepatitis C virus
CHC: Chronic hepatitis C
IFN-α: Interferon α
BDI: Beck Depression Inventory
PSE: Present state examination
MADRS: Montgomery Åsberg Depression Rating Scale
IL-6: Interleukin 6
